# Electrochemical Corrosion Behavior of Ni-Fe-Co-P Alloy Coating Containing Nano-CeO_2_ Particles in NaCl Solution

**DOI:** 10.3390/ma12162614

**Published:** 2019-08-16

**Authors:** Xiuqing Fu, Wenke Ma, Shuanglu Duan, Qingqing Wang, Jinran Lin

**Affiliations:** 1 College of Engineering, Nanjing Agricultural University, Nanjing 210095, China; 2 Key laboratory of Intelligence Agricultural Equipment of Jiangsu Province, Nanjing 210031, China

**Keywords:** scanning electrodeposition, Ni-Fe-Co-P-CeO_2_ composite coating, electrochemical corrosion behavior, corrosion mechanism

## Abstract

In order to study the effect of nano-CeO_2_ particles doping on the electrochemical corrosion behavior of pure Ni-Fe-Co-P alloy coating, Ni-Fe-Co-P-CeO_2_ composite coating is prepared on the surface of 45 steel by scanning electrodeposition. The morphology, composition, and phase structure of the composite coating are analyzed by means of scanning electron microscope (SEM), energy dispersive spectroscopy (EDS), and X-ray diffraction (XRD). The corrosion behavior of the coatings with different concentrations of nano-CeO_2_ particles in 50 g/L NaCl solution is studied by Tafel polarization curve and electrochemical impedance spectroscopy. The corrosion mechanism is discussed. The experimental results show that the obtained Ni-Fe-Co-P-CeO_2_ composite coating is amorphous, and the addition of nano-CeO_2_ particles increases the mass fraction of P. With the increase of the concentration of nano-CeO_2_ particles in the plating solution, the surface flatness of the coating increases. The surface of Ni-Fe-Co-P-1 g/L CeO_2_ composite coating is uniform and dense, and its self-corrosion potential is the most positive; the corrosion current and corrosion rate are the smallest, and the charge transfer resistance is the largest, showing the best corrosion resistance.

## 1. Introduction

Corrosive environments are one of the most common service environments for metal components in engineering. Due to the high chemical activity of Fe in such environments, the engineering application of steel components therein is facing severe challenges due to insufficient corrosion resistance [1,2]. Surface modification is one of the most effective ways to solve this problem. The electroplating process for the preparation of nanocomposites is a process for the co-deposition of nanoparticles and metal ions on the surface of a cathode workpiece via the electrochemical principle, and a process to obtain nanocomposites that demonstrate superior performance [3,4]. Scanning electrodeposition technology, as an extension of electroplating technology, is widely used in machinery, aerospace, electronics industry, etc., due to its controllability, high efficiency, selectivity, and superior coating performance [5,6]. In recent years, many scholars have devoted to improving the performance of traditional nickel-based alloy coatings. Usually, tungsten [7], copper [8], iron [9], cobalt [10], and other metal ions [11] are introduced into the electrolyte, thereby processing a multi-component alloy. The multi-component overcomes the shortcomings of unary and binary alloy coatings, and has good wear resistance and corrosion resistance, which meet the varying performance requirements of composite materials [12,13].

It has been found in research that the application properties and functions of alloy coating can be further improved by co-depositing second phase nano-oxide particles in a nickel-based alloy coating [14,15]. The rare earth element cerium is the only stable tetravalent element. It has a unique oxidizing property and a large effective nuclear charge number, which can catalyze many reactions, and is widely used in various applications. Cerium oxide is a typical rare earth oxide with good wear resistance and corrosion resistance, and can be used as a nanoparticle reinforcement phase in various applications [16,17,18]. In order to further improve the corrosion resistance of traditional nickel-based coatings, Ni-Fe-Co-P-CeO_2_ composite coatings are prepared by scanning electrodeposition technology. The concentration of nano-CeO_2_ particles in the plating solution is applied to the coating of Ni-Fe-Co-P alloys. The influence of appearance and structure, and the electrochemical corrosion behavior of composite coatings, provide a reference for the development of new composite materials.

## 2. Materials and Methods

### 2.1. Experimental Principle

**Figure 1:**
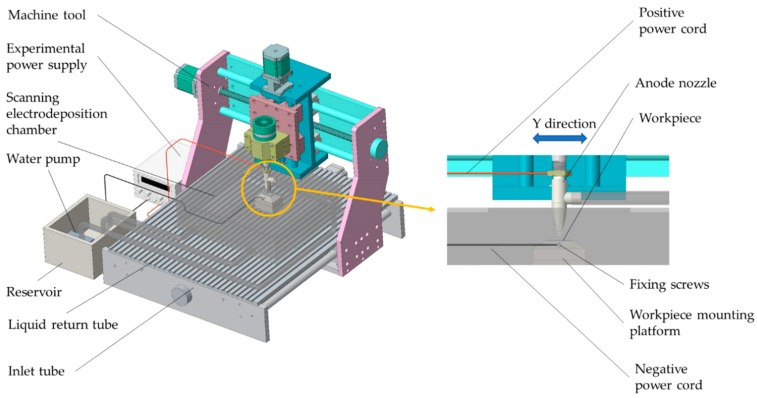
Scanning electrodeposition test device.

The scanning electrodeposition test apparatus is shown in Figure 1, wherein the anode nozzle is mounted on the machine tool spindle; the workpiece is mounted on the workpiece mounting platform by tightening the fixing screws. During the scanning electrodeposition process, the anode bed of the anode nozzle reciprocates in the Y direction, and the water pump presses the plating solution from the reservoir into the anode nozzle through the inlet tube and sprays it on the surface of the workpiece at high speed to spray the plating solution in the electrodeposition chamber. The liquid return tube flows back to the reservoir to realize the circulation of the plating liquid. After the power is turned on, the plating solution sprayed on the surface of the workpiece through the anode nozzle forms a closed loop, and under the action of the external electric field, a redox reaction occurs to realize deposition of metal ions. The scanning length during the test is 20 mm, and the scanning speed is 13.5 mm/s. The height between the bottom of the anode nozzle and the workpiece processing surface is 1.5 mm.

### 2.2. Materials and Methods

Fourty five steel with dimensions of 25 mm × 10 mm × 8 mm was used as substrate material, and its chemical composition is listed in Table 1. Table 2 shows the formulation of the plating solution used. The drugs used are of analytical grade and are prepared with deionized water. The particle size of the nano-CeO_2_ particles in the test was 100 nm, and the concentration of nano-CeO_2_ particles in the plating solution was 0, 0.5, 1, and 1.5 g/L, respectively. The cathode workpieces are polished with 800# and 1500# water sandpaper, respectively, and the workpiece is pretreated before scanning electrodeposition process; the specific process is shown in the Table 3, after each step is rinsed with deionized water. The workpiece that has been subjected to the pre-treatment is placed in a spray electrodeposition test apparatus for a sputtering test. The current during the spray electrodeposition process is 0.6 A, the pH of the plating solution is 1.0–1.5, the bath temperature is 60 °C, and the plating time is 20 min. After the end of the scanning electrodeposition process, the workpiece was subjected to ultrasonic cleaning and drying treatment, and performance studies were performed.

### 2.3. Characterization

The morphology of the coating was observed by scanning electron microscopy (FEI-SEM, Quanta FEG250; FEI Instruments, Hillsboro, OR, USA), with an accelerating voltage of 15 kV and image type of secondary electron image (SEI); the chemical composition of the coating was determinated by energy dispersive spectroscopy (EDS, XFlash 5030 Bruker AXS, Inc., Berlin, Germany), with an accelerating voltage of 16 kV and the working distance of 11 mm; the phase structure of the coating was analyzed by X-ray diffraction (XRD, PANalytical X’pert; PANalytical Inc., Almelo, The Netherlands), with a radiation source of Cu Kα (λ = 0.15405 nm), operating voltage of 40 kV, scan rate of 5 °/min, and scanning range (2θ) of 10°~80°, using HighScore Plus 3.0 to analyze the results.

**Figure 2:**
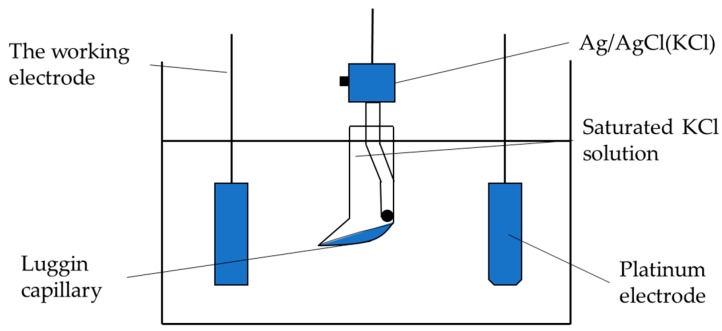
Electrochemical detection device schematic.

The corrosion resistance of the coating was detected by electrochemical test of the three-electrode system (Figure 2). The working electrode is the workpiece, and the auxiliary electrode is Pt piece; the reference electrode is saturated calomel electrode (SCE), and the Tafel polarization curve measurement and electrochemical impedance spectroscopy (EIS) are completed by electrochemical workstation CS350 (Wuhan Corrtest Instruments Corp., Ltd., Wuhan, China). In the test, the workpiece to be tested was encapsulated with epoxy resin and immersed in a 50 g/L NaCl solution, and the Tafel polarization curve of the coating was obtained by a potentiodynamic scanning method and then obtained by polarization curve epitaxy. Corrosion potential, corrosion current, and other parameters were used to explore the corrosion resistance of the coating and the substrate. Under the open circuit potential, the impedance spectrum of the coating in NaCl solution was tested by the alternating current impedance method (EIS). The test frequency was 0.01–10^5^ Hz, and the scanning direction was from high frequency to low frequency. The impedance fitting of different coatings was performed by Zview 2 software analysis.

## 3. Results

### 3.1. Coating Morphology Analysis and Composition

**Figure 3:**
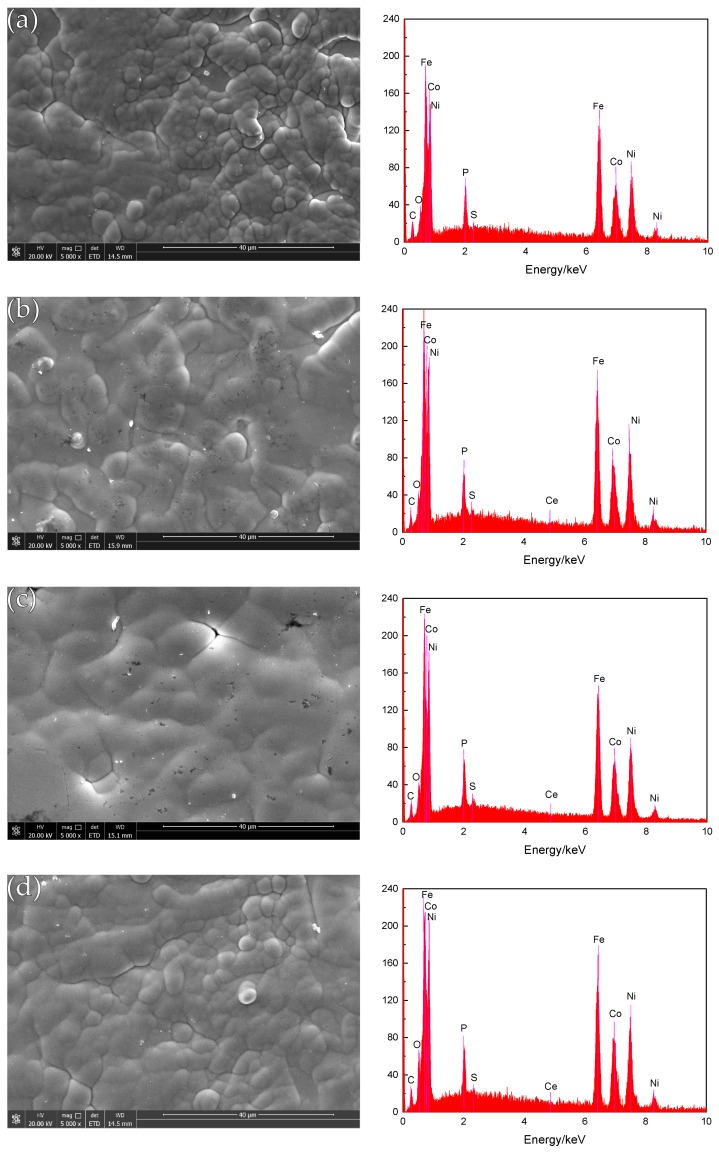
Surface morphology of the coatings before corrosion and EDS spectrum of coatings: (**a**) Ni-Fe-Co-P; (**b**) Ni-Fe-Co-P-0.5g/L CeO_2_; (**c**) Ni-Fe-Co-P-1g/L CeO_2_; and (**d**) Ni-Fe-Co-P-1.5g/L CeO_2_.

The SEM photographs in Figure 3 (image type is SEI) show the surface topography of the composite coating before corrosion. It can be seen that before the corrosion, the coating structure of different nano-CeO_2_ particles is composed of different sizes of cells, the arrangement is tight, and no obvious defects are found. When the concentration of nano-CeO_2_ particles is 0 g/L (Figure 3a), the cytoplasm is a spherical hillock-like structure, but the size difference is large, and there are also defects such as pores and protrusions. When a small number of nano-CeO_2_ particles is added to the plating solution (Figure 3b), the surface flatness of the coating is improved, but the cell structure has partial protrusions, the boundary is tortuous, and there are some defects such as pores. When the concentration is increased to 1 g/L (Figure 3c), the surface of the coating is dense and flat, the structure is compact, the cells are closely arranged, and the boundary is very blurred, and there are no obvious protrusions and impurity pores. When the concentration of nano-CeO_2_ particles in the plating solution reaches 1.5 g/L or more (Figure 3d), the surface morphology of the coating can be seen to have obvious agglomeration, and the surface of the coating is rough and uneven, with protrusions and defects generated. According to the analysis, the scanning jet of the plating solution accelerates the ion transport, increases the limiting current density, and strengthens the cathodic polarization, so that the deposition is performed at a high flow density [5]. The formation process of the compact nickel-based coating is similar to that of soil plant growth, and the nano-CeO_2_ particles dispersed in the plating solution are similar to the seeds, and are adsorbed on the surface of the substrate by tiny solid particles, because the rare earth element Ce is the third sub-group element. It has a large effective charge number and exhibits strong adsorption capacity. It can adsorb Ni^2+^, Fe^2+^, Co^2+^, and other ions [5]. As the deposition progresses, the seeds gradually grow, forming a cell structure with many different sizes. When the nano-CeO_2_ particles are excessive, they are excessively adsorbed on the surface of the metal substrate, causing the surface-active sites of the matrix to be masked and lose their activity, thereby greatly reducing or even inhibiting the nucleation sites, and uneven nanoparticle agglomerates are deposited on the surface of the plating layer. The formation of larger protrusions affects the quality of the coating, and the advantage of nano-CeO_2_ particles is not obvious.

**Figure 4:**
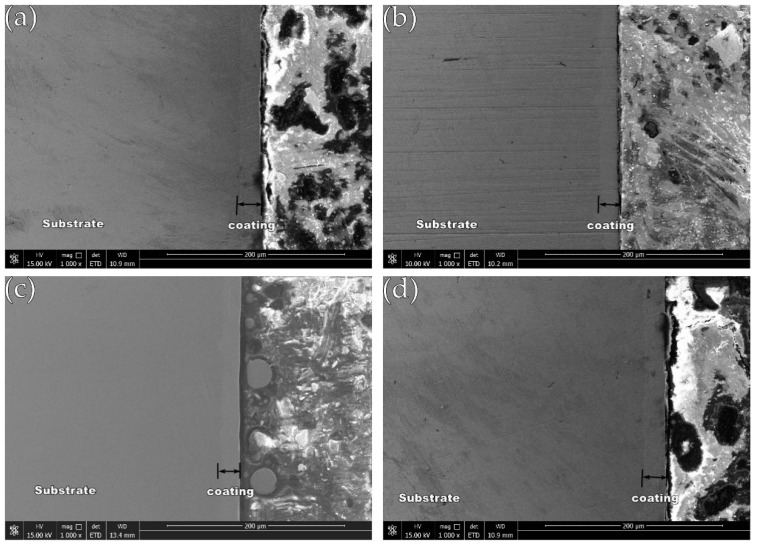
Cross-section morphology of the coatings: (**a**) Ni-Fe-Co-P; (**b**) Ni-Fe-Co-P-0.5 g/L CeO_2_; (**c**) Ni-Fe-Co-P-1 g/L CeO_2_; and (**d**) Ni-Fe-Co-P-1.5 g/L CeO_2_.

After cutting and inlaying the test piece, the cross-section of the test piece is observed by SEM, and the cross-sectional shape of the obtained coating is shown in Figure 4 (image type is SEI). It is obvious that the Ni-Fe-Co-P-CeO_2_ composite coating is uniform and dense, and there are no larger defects such as cracks and holes, effectively shielding the corrosion passage of the corrosive medium into the substrate and retarding the corrosion.

**Figure 5:**
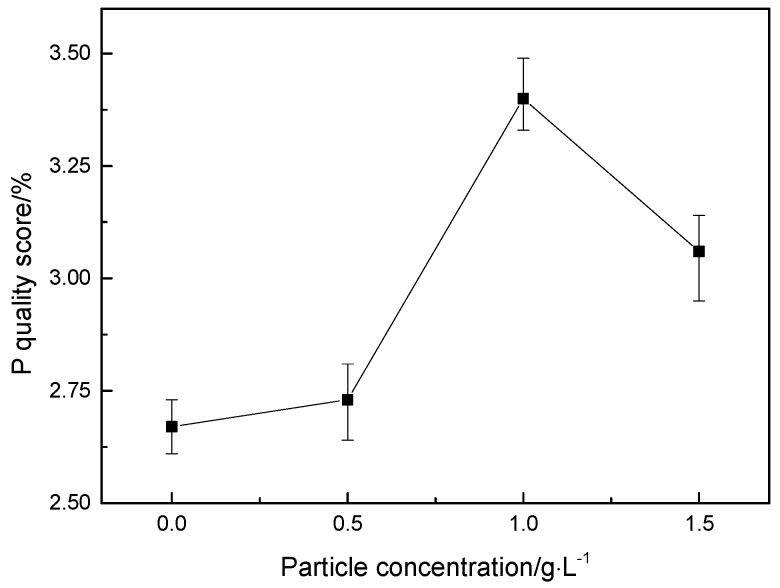
P mass fraction of coatings with different concentration of nano-CeO_2_ particles.

Using EDS technology, the EDS spectrum obtained by analyzing the composition of the surface of the coating is shown in Figure 3. Ni, Fe, Co, and P elements are present in all the energy spectra, and an appropriate number of nano-CeO_2_ particles are added to the plating solution. The energy spectrum of the surface of the coating shows a slight peak of Ce element (Figure 3b–d), which indicates that the prepared coating is a quaternary Ni-Fe-Co-P alloy coating and Ni-Fe-Co-P-CeO_2_ composite coating. Figure 5 shows the mass fraction of P element in the coatings of different nano-CeO_2_ particles obtained by EDS analysis. It can be seen that the mass fraction of P element increases first and then decreases with the increase of the concentration of nano-CeO_2_ particles, and when the concentration of nano-CeO_2_ is 1 g/L, the maximum value is 3.40%. Since P element will be enriched and hydrolyzed on the surface of the electrolyte to form hypophosphite, a phosphorus-rich film is formed between the coating and the interface of the corrosive medium to make the nickel-based coating exhibit high corrosion resistance. Adding an appropriate number of nano-CeO_2_ particles to the plating solution increases the P content in the coatings. The increase of the P element content shortens the film formation time of the phosphating film on the surface of the coatings, and also increases the thickness of the phosphating film, which contributes to the improvement of the corrosion resistance of the coatings [19,20].

**Figure 6:**
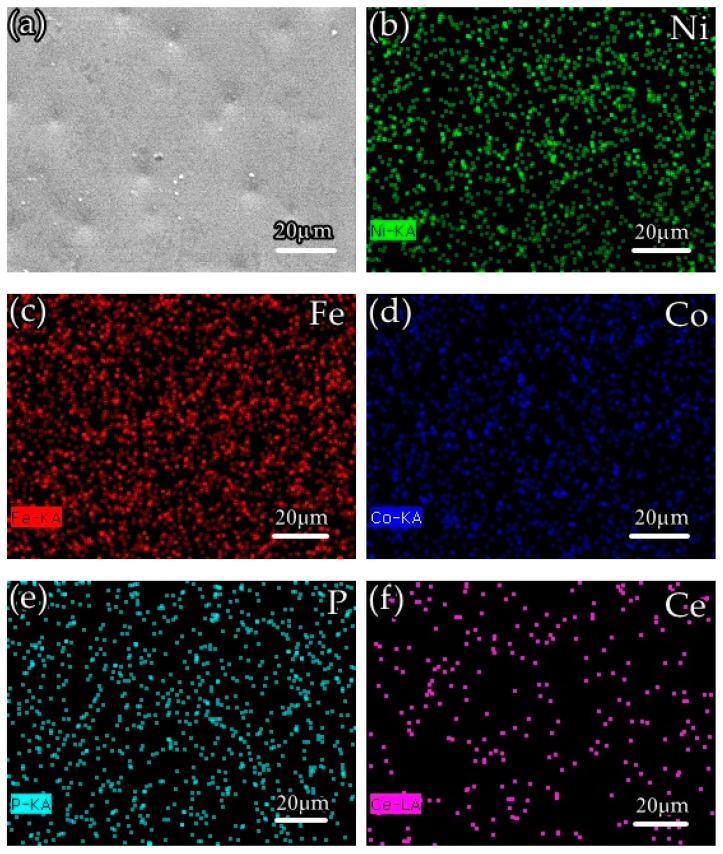
Elemental surface mapping of Ni-Fe-Co-P-1 g/L CeO_2_ composite coating:(**a**) the SEM image of the analyzed surface (**b**) Ni content; (**c**) Fe content; (**d**) Co content; (**e**) P content; and (**f**) Ce content.

Figure 6 shows an elemental view of the surface of the Ni-Fe-Co-P-1 g/L CeO_2_ composite coating, wherein the Ce element diagram (Figure 6f) represents nano-CeO_2_ particles, and it can be seen that the alloying elements and the nano-CeO_2_ particles are uniformly distributed on the surface of the plating layer. Studies have shown that the uniform distribution of elements and particles is due to the improved corrosion resistance of the coating.

### 3.2. Plating Phase Structure

**Figure 7:**
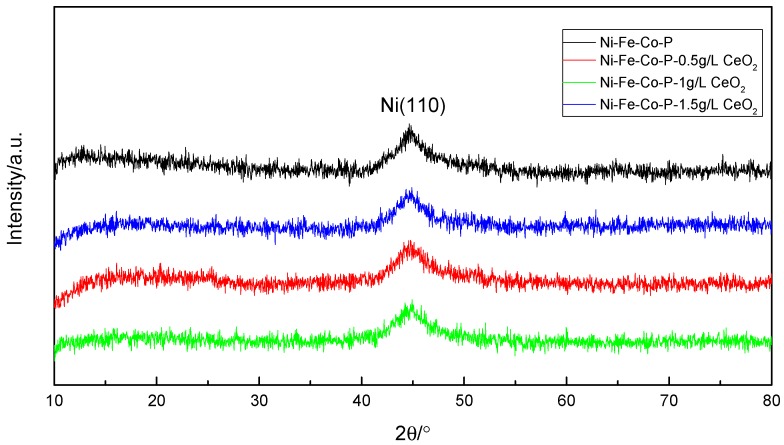
XRD patterns of coatings with different concentration of nanometer CeO_2_ particles.

Figure 7 is an XRD pattern of the coating obtained by X-ray diffraction test. It can be seen that the coating is a typical amorphous structure, and there is a significant diffuse scattering broadening peak (Ni (110)) between 42° and 48° in 2θ. The peak width of the diffraction peak of the nanocrystalline alloy coating did not produce obvious changes, and peak intensity changes were not obvious, indicating that the nano-CeO_2_ particles did not obviously change in the phase structure of Ni-Fe-Co-P coating. For the nickel-phosphorus coating, the crystal structure depends mainly on the P element content in the coating. The authors of [21] have shown that when P content is lower than 5%, it is usually crystalline structure, and when P content is higher than 6.5%, it becomes amorphous structure. In this test, since the prepared plating layer is a quaternary alloy plating layer, and the atomic structure, size, and electronegativity of Ni, Fe, Co, and P elements are largely different, the amorphous forming ability is enhanced. Therefore, the plating layer is still amorphous when the P content is low. It is generally believed that the amorphous coating has better corrosion resistance due to the absence of local electrochemical potential difference between crystal grains and grain boundaries in the crystalline coating [18].

### 3.3. Tafel Polarization Curve

**Figure 8:**
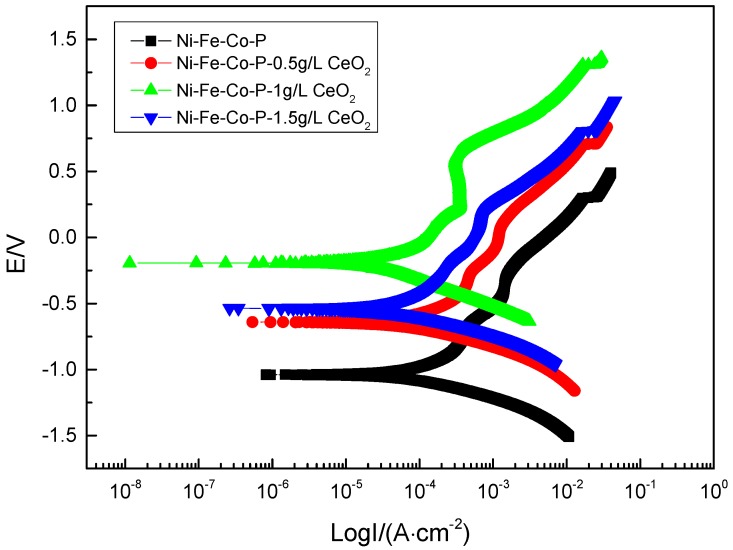
Polarization curves of coatings with different concentrations of nanometer CeO_2_ particles.

Figure 8 shows the polarization curves of the composite coatings in the 50 g/L NaCl solution. The corrosion parameters obtained by Cview 2 software and polarization curve epitaxy are shown in Table 4. It can be seen from Figure 8 that the anodic polarization process of the composite coating is hindered and a significant passivation behavior occurs, and the composite coating is obtained when the concentration of nano-CeO_2_ particles in the plating solution is 1 g/L. The passivation zone is significantly larger than the remaining composite coating. It can be seen from Figure 8 and Table 4, compared with the polarization curve of pure Ni-Fe-Co-P alloy coating, that the polarization curve of composite coating prepared by co-deposition of a certain number of nano-CeO_2_ particles by scanning electrodeposition technology moves up and left as a whole. With the increase of the concentration of nano-CeO_2_ particles in the plating solution, the self-corrosion potential is continuously shifted, and the corrosion current density is gradually reduced. When the concentration of nano-CeO_2_ particles is 1 g/L, the prepared Ni-Fe-Co-P-CeO_2_ composite coating has the most positive self-corrosion potential (−0.19372 V) and the minimum corrosion current density (1.5375 × 10^−5^ A·cm^−2^). While continuing to increase the concentration of nano-CeO_2_ particles, the corrosion potential is negatively shifted, and the corrosion current density is significantly increased, indicating that corrosion resistance has begun to decline. According to the principle of corrosion electrochemistry, the larger the corrosion potential is, the smaller the corrosion current density is, the smaller the corrosion tendency of the material is, and the better the corrosion resistance is. Therefore, the concentration of nano-CeO_2_ particles is 1 g/L. The Ni-Fe-Co-P-CeO_2_ composite coating has the best corrosion resistance. Studies have shown that anodic polarization can slow metal corrosion, and the degree of anodic polarization directly affects the speed of the anode process [22]. Compared with the pure Ni-Fe-Co-P coating, the addition of nano-CeO_2_ particles increases the hindrance of the corrosion process of the nickel-based coating. The Ba and Bc of the polarization curve of the composite coating are increased compared with the coating of the undoped nano-CeO_2_ particles; especially, the blocking effect (Ba) of the anode is more significant. When excessive nano-CeO_2_ particles are added to the plating solution, too much rare earth oxide adsorbs on the surface of the substrate, hindering the adsorption of Ni, Co, and Fe element on the surface of the substrate, which hinders the deposition of particles, which is not conducive to the plating. The formation of its corrosion resistance has been weakened.

### 3.4. Analysis of Electrochemical Impedance Spectroscopy

**Figure 9:**
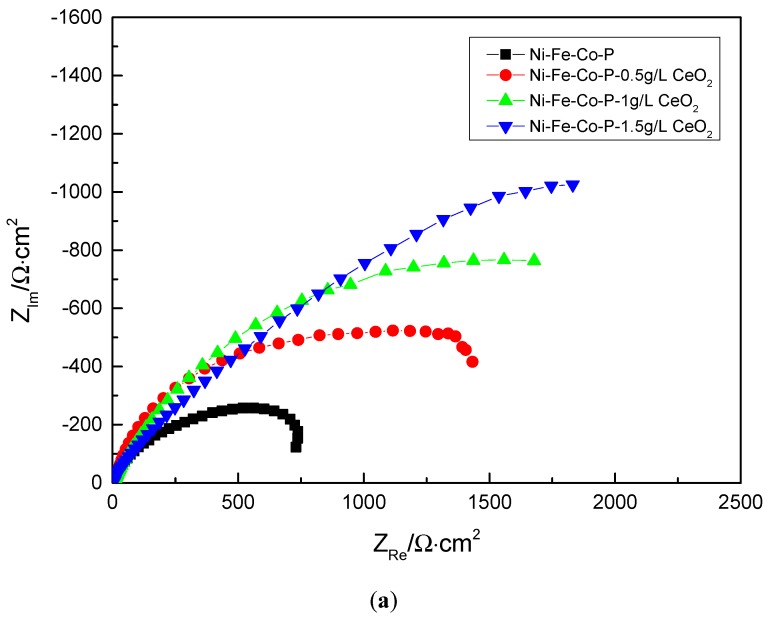

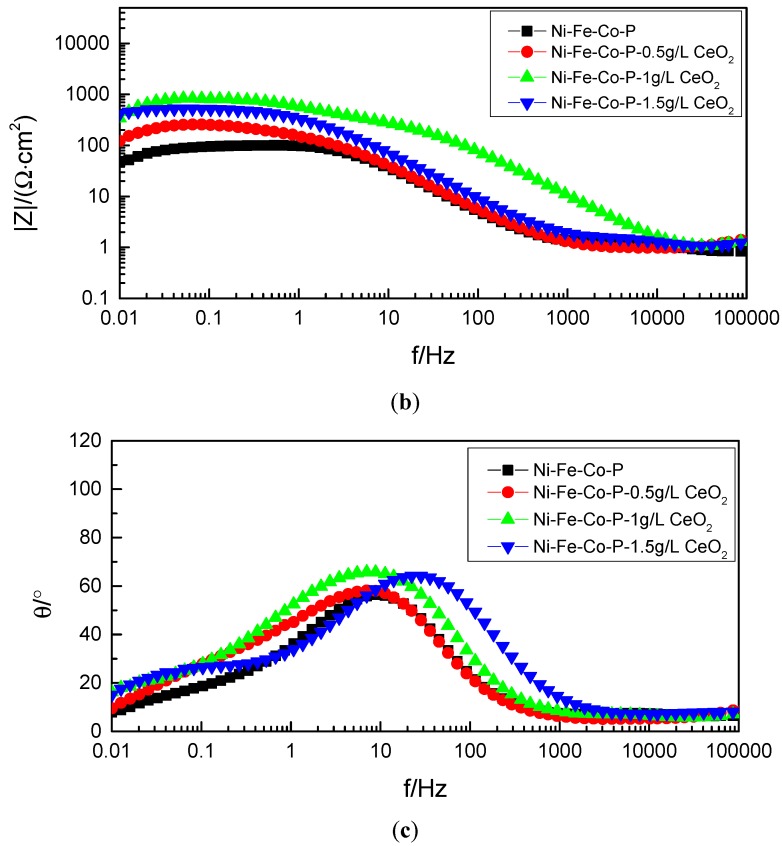
Alternating current impedance method EIS of coatings with of coatings with different concentrations of nanometer CeO_2_ particles: (**a**) Nyquist diagram, (**b**) Bode diagram—impedance curve, and (**c**) Bode diagram—phase Angle curve.

**Figure 10:**
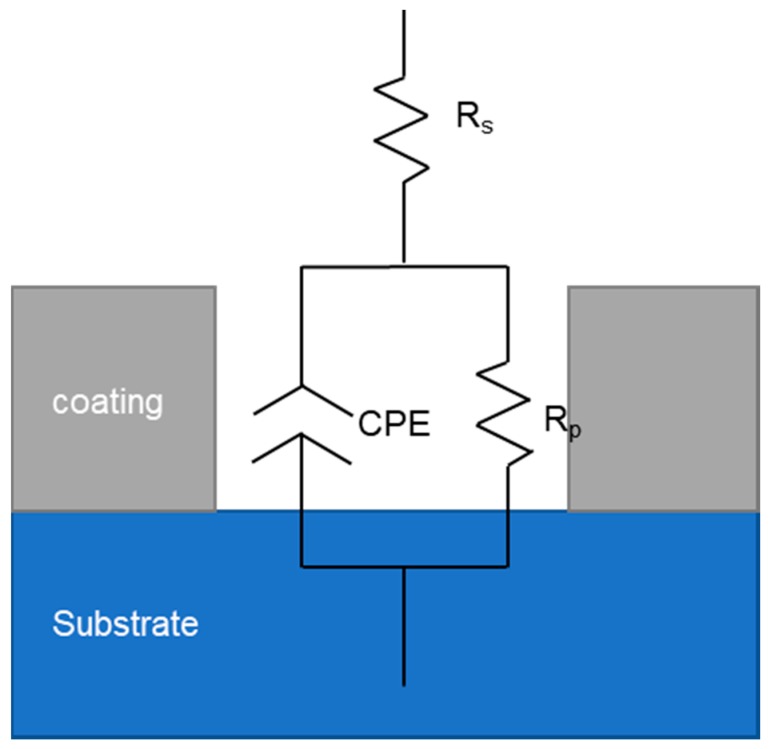
Equivalent circuit diagram.

In order to further explore the mechanism of electrochemical corrosion of Ni-Fe-Co-P-CeO_2_ composite coating, more corrosion kinetic information is obtained. The AC impedance analysis of the composite coating is performed under open circuit potential. The electrochemical impedance spectrum obtained in Figure 9 is shown. The Nyquist diagram of the composite coating (Figure 9a) shows a single capacitive reactance arc characteristic, and the Bode diagram (Figure 9c) has only one peak, indicating that the time constant is 1, and the electrode reaction process is mainly affected by the charge [23]. The transfer effect also indicates that the corrosive medium only contacts the interface of the coating and does not penetrate into the surface of the substrate due to diffusion. It can be seen from the phase angle curve of the Bode diagram (Figure 9c) that the maximum phase angle of the Ni-Fe-Co-P-CeO_2_ composite coating is higher than that of the pure nickel-based coating (56.379). From the impedance curve (Figure 9b), the impedance modulus of the composite coating doped with nano-CeO_2_ particles is higher than that of the undoped nano-CeO_2_ particles throughout the scanning frequency interval. This shows that the corrosion resistance of the Ni-Fe-Co-P alloy coating is effectively improved by co-depositing nano-CeO_2_ particles. It can also be seen from the Nyquist diagram of Figure 9 (Figure 9a) that the radius of the capacitive reactance of the Ni-Fe-Co-P-CeO_2_ composite coating is much larger than that of the Ni-Fe-Co-P alloy coating. When the concentration of nano-CeO_2_ particles is 1 g/L, the radius of the capacitive anti-arc is the largest, and the radius of the capacitive anti-arc is used as the characterization of the corrosion resistance of the coating. The larger the radius, the greater the resistance of charge transfer and the harder the corrosion reaction. This result shows that the Ni-Fe-Co-P-CeO_2_ composite coating has better corrosion resistance. The AC impedance spectrum is modeled by the equivalent circuit diagram shown in Figure 10 and fitted by Zview software. The obtained fitting data is shown in Table 5. In the equivalent circuit diagram, Rs is the resistance in the solution. Rp is a charge transfer resistor, CPE is a constant phase angle element, and its impedance is

$$Z=1/Y_{0}{\left(j\omega \right)}^{-n}$$

its type has two parameters: constant Y_0_, its dimension is Ω^−1^·cm^−2^·s^−n^; parameter n, dimensionless index. When n = 1, the CPE component is the ideal capacitor. When n = 0, the CPE component is pure resistance, and in the actual solution, n is between 0 and 1 [7]. Obviously, with the addition of nano-CeO_2_ particles, the charge transfer resistance of the composite coating increases first and then decreases but the charge transfer resistor (R_p_) of the doped nano-CeO_2_ particles is always larger than that of the pure nickel-based coating, and the corrosion resistance is extremely high great improvement. When the concentration of nano-CeO_2_ particles in the plating solution is too large, the nano-CeO_2_ particles are easily agglomerated, and the inclusions formed are increased, resulting in loose coating structure, and the strengthening effect of the nano-CeO_2_ particles is weakened.

### 3.5. Surface Morphology after Corrosion of the Coating

**Figure 11:**
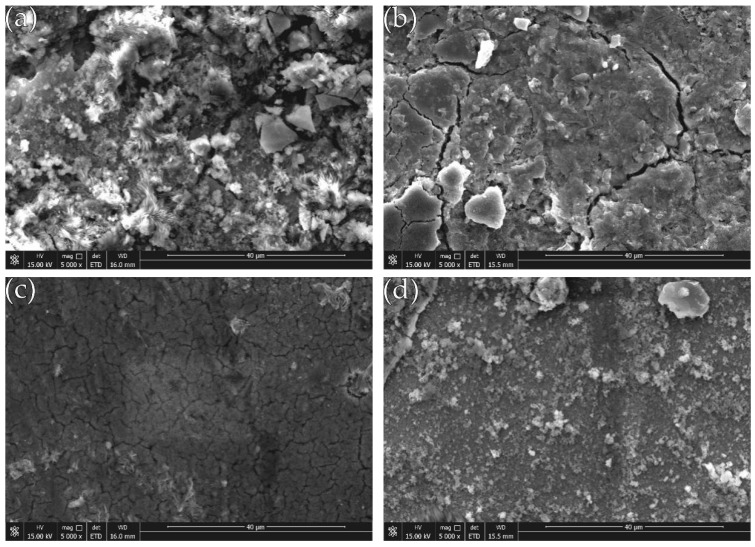
Surface morphology after plating corrosion: (**a**) Ni-Fe-Co-P; (**b**) Ni-Fe-Co-P-0.5 g/L CeO_2_; (**c**) Ni-Fe-Co-P-1 g/L CeO_2_; and (**d**) Ni-Fe-Co-P-1.5 g/L CeO_2_.

The SEM photographs in Figure 11 (image type is SEI) shows the surface topography of the composite coating after corrosion. It can be seen that after 5 days of etching in 50 g/L NaCl solution, many micro-protrusions of different sizes appear on the surface of the coating, and the surface appears more frequently black corrosion product. A large number of narrow and shallow microcracks extend along the boundaries of the cell structure. The degree of corrosion of the composite coating with different concentrations of nano-CeO_2_ particles in the plating solution is different. Among them, the coating of undoped nano-CeO_2_ particles (Figure 11a) is most corroded, and a large amount of corrosion product is deposited on the surface. When the concentration of nano-CeO_2_ particles is 1 g/L (Figure 11c), the coating is the least corroded and has a stronger retarding effect on corrosive media.

Generally speaking, the corrosion of metal in NaCl solution is mainly due to the presence of Cl^−^, the Cl^−^ radius is small, the penetrating ability is very strong, and the adsorption is unevenly in the vicinity of the boundary and the impurity, so that the local dissolution is dominant, and pitting micropores are formed. Even if the surface has a passivation film formed by the metal, Cl^−^ can form a soluble compound with the cation of the passivation film, destroying the dynamic balance of dissolution and repair of the passivation film, causing the passivation film to be gradually eliminated and continue to be corroded, resulting in etching. The deepening of the hole can quickly become a corrosion pit. When sprayed electrodeposition is used to prepare Ni-Fe-Co-P alloy coating, it is always accompanied by hydrogen evolution reaction, which retards the discharge deposition of Ni, Co, and Fe elements, which leads to the formation of pinholes or pits on the surface of the coating. In addition, the transition elements are sprayed. During the electrodeposition process, the amount of hydrogen absorption is large. The hydrogen atoms that penetrate into the cell by diffusion will cause distortion of the cytoplasm, forming a large internal stress, and stress corrosion occurs during the corrosion process. After the corrosion, the surface of the plating layer is easily cracked [24]. Since the deposition process of the sprayed electrodeposited Ni-Fe-Co-P alloy coating is a process of uneven reduction and accumulation of Ni, Co, Fe, and P, the atomic size is different and the arrangement is different, which can only be disorderly stacked and reflected to the plating layer [25]. On the top, the cell material is relatively dispersed, which provides conditions for the diffusion of corrosive media, but the corrosion condition after the addition of nano-CeO_2_ particles is improved. The reason is: first, the filling effect of the nano-CeO_2_ particles between the coating boundaries makes the structure of the composite coating is more uniform and dense, the porosity is greatly reduced, and the rare earth elements have strong affinity with impurity elements such as O and H, and these impurity elements can be wrapped to form a rare earth composite phase, and the surface of the coating is dispersed. The composite phase coverage causes the permeation channel of Cl^−^ ions to be effectively intercepted, thereby enhancing the corrosion resistance of the composite coating [26]. Secondly, because the potential of nano-CeO_2_ particles is in Ni, Co, and Fe metals, it is easy to form microscopic galvanic cells at the interface between nano-CeO_2_ particles and nickel-based alloy. Nano-CeO_2_ particles are used as the cathode, and Ni, Co, and Fe are the anode. This galvanic reaction changes the coating from local spot corrosion to uniform corrosion, which helps to slow down the corrosion. When the concentration of nano-CeO_2_ particles is too large, the metal ions are precipitated in a large amount as a complex, and the amount of precipitation increases, but the actual deposition rate decreases, and the corrosion tendency of the coating increases [27].

## 4. Conclusions

In this paper, a Ni-Fe-Co-P-CeO_2_ composite coating was prepared using the scanning electrodeposition technique. To explore the impact of the concentration of nano-CeO_2_ particles in the plating solution on the micro morphology, structure, and composition of the coating, and to study the strengthening mechanism of nano-CeO_2_ particles on the electrochemical corrosion behavior of Ni-Fe-Co-P alloy coating, the following conclusions were drawn:

(1) The surface structure of Ni-Fe-Co-P-CeO_2_ composite coating is dense, with fewer defects, and the bonding between the coating and the substrate is good. The addition of nano-CeO_2_ particles increases the P mass fraction of the coating, which helps slow down corrosion.
(2) The Ni-Fe-Co-P-CeO_2_ composite coating is still amorphous in the case of low P mass fraction.
(3) The Ni-Fe-Co-P-1 g/L CeO_2_ composite coating has the most positive self-corrosion potential, the lowest self-corrosion current density, and the best corrosion resistance.
(4) With the increase of the concentration of nano-CeO_2_ particles in the plating solution, the impedance spectrum of Ni-Fe-Co-P-CeO_2_ composite coating is nonlinearly related to the charge-transfer resistance of the equivalent circuit, which increases first and then decreases. Regularly, the Ni-Fe-Co-P-1 g/L CeO_2_ composite coating has the largest charge transfer resistance (2941 Ω·cm^−2^) and the weakest corrosion tendency.
(5) After corrosion, micro-cracks and a large number of corrosion products appear on the surface of the coating. After doping with appropriate number of nano-CeO_2_ particles, the alloy coating can inhibit this corrosion, and the corrosion degree of the Ni-Fe-Co-P-1 g/L CeO_2_ composite coating is the smallest, showing the best corrosion resistance.

## Figures and Tables

**Table 1:** Chemical composition of 45 steel (mass fraction).

C	Si	Mn	Cr	Ni	Cu
0.42~0.50%	0.17~0.37%	0.50~0.80%	≤0.25%	≤0.30%	≤0.25%

**Table 2:** Composition of plating solution.

Plating Solution Composition	Content (g/L)
Nickel sulfate hexahydrate (NiSO_4_·6H_2_O)	120
Nickel chloride hexahydrate (NiCl_2_·6H_2_O)	40
Ferrous sulfate (FeSO_4_·7H_2_O)	20
Cobalt chloride (CoCl_2_·6H_2_O)	10
Phosphoric acid (H_3_PO_3_)	30
Orthoboric acid (H_3_BO_3_)	30
Citric acid (C_6_H_8_O_7_)	10
Thiourea (CH_4_N_2_S)	0.01
Sodium dodecyl sulfate (C_12_H_25_SO_4_Na)	0.08

**Table 3:** The process of workpiece pretreatment.

Step	Solution Formula	Content (g/L)	Process Parameters
Electric net degreasing	Sodium hydroxide (NaOH)	25	Current = 1 A Power-on time = 20 s pH = 13
	Sodium carbonate (Na_2_CO_3_)	21	
	Trisodium phosphate anhydrous (Na_3_PO_4_)	50	
	Sodium chloride (NaCl)	2	
Weak activation	Hydrochloric Acid (HCl)	25	Current = 1 A Power-on time = 30 s pH = 0.3
	Sodium chloride (NaCl)	140	
Strong activation	Trisodium citrate dihydrate (Na_3_C_6_H_5_O_7_·2H_2_O)	140	Current = 1 A Power-on time = 20 s pH = 4
	Citric acid (C_6_H_8_O_7_)	94	
	Nickel chloride hexahydrate (NiCl_2_·6H_2_O)	3	

**Table 4:** Composition of plating solution.

Sample	Ba (mV)	Bc (mV)	I_corr_ (A·cm^−2^)	E_corr_ (V)	Error (%)
Ni-Fe-Co-P	156.7	155.8	8.0989 × 10^−5^	−1.0394	5.96
Ni-Fe-Co-P-0.5 g/LCeO_2_	243.09	156.47	6.6569 × 10^−5^	−0.64157	6.14
Ni-Fe-Co-P-1 g/LCeO_2_	336.01	174.46	1.5375 × 10^−5^	−0.19372	7.01
Ni-Fe-Co-P-1.5 g/LCeO_2_	246.58	244.66	4.5404 × 10^−5^	−0.5361	9.93

**Table 5:** Equivalent circuit diagram parameter value.

Sample	R_s_ (Ω·cm^−2^)	CPE-T (F·cm^−2^)	CPE-P	R_p_ (Ω·cm^−2^)	Error (%)
Ni-Fe-Co-P	10.47	0.0011242	0.73659	776.1	4.46
Ni-Fe-Co-P-0.5 g/L CeO_2_	11.31	0.000526	0.79027	1513	3.12
Ni-Fe-Co-P-1 g/L CeO_2_	2.229	0.00054346	0.62269	2941	4.30
Ni-Fe-Co-P-1.5 g/L CeO_2_	2.807	0.00084912	0.60494	2631	6.85
